# Safety of Vortioxetine in Patients With Depression: A Post‐Marketing Surveillance Study of Intracranial Hemorrhage in a Japanese Health Insurance Claims Database

**DOI:** 10.1002/npr2.70077

**Published:** 2025-12-25

**Authors:** Takeshi Inoue, Rie Otake, Tatsuya Hoshino

**Affiliations:** ^1^ Department of Psychiatry Tokyo Medical University Tokyo Japan; ^2^ Department of Psychiatry Sapporo Hanazono Hospital Sapporo Japan; ^3^ Japan Medical Office Takeda Pharmaceutical Company Ltd Tokyo Japan

**Keywords:** depression, gastrointestinal hemorrhage, intracranial hemorrhage, selective serotonin reuptake inhibitors, vortioxetine

## Abstract

**Aim:**

As part of a post‐marketing commitment, this study evaluated the incidences of intracranial hemorrhage and serious (intracranial or gastrointestinal) hemorrhage requiring hospitalization in Japanese patients with depression prescribed vortioxetine or selective serotonin reuptake inhibitors (SSRIs).

**Methods:**

This real‐world, retrospective cohort study used data from the JMDC claims database. Patients with depression who received an initial prescription for vortioxetine or SSRIs (index date) between November 2019 and November 2022, with an observation period of 6 months before (look‐back period) and up to 360 days after (follow‐up period) the index date were included. The primary analysis estimated the incidence of intracranial hemorrhage, while the secondary analysis estimated the incidence of serious hemorrhage requiring hospitalization. A multivariate model incorporating age, sex, antithrombotic and nonsteroidal anti‐inflammatory drug prescriptions, and hypertension as covariates was used to calculate adjusted hazard ratios (aHRs). A propensity score incorporating 21 additional covariates assessed the robustness of the primary analysis (sensitivity analysis).

**Results:**

Overall, 147 777 patients were included; 22 827 were prescribed vortioxetine (exposure) and 124 950 were prescribed SSRIs (control). The incidence of intracranial hemorrhage was 1.5 per 10 000 person‐years in the exposure group and 3.2 per 10 000 person‐years in the control group (aHR: 0.5, 95% confidence interval [CI]: 0.1–1.9). The incidence of serious hemorrhage requiring hospitalization was 27.4 per 10 000 person‐years in the exposure group and 31.4 per 10 000 person‐years in the control group (aHR: 0.8, 95% CI: 0.6–1.2). The incidence of intracranial hemorrhage in the exposure group versus the control group in the sensitivity analysis was similar to the primary analysis (aHR: 0.4, 95% CI: 0.1–1.9).

**Conclusion:**

The incidence of intracranial hemorrhage and serious hemorrhage requiring hospitalization was low in Japanese patients with depression prescribed vortioxetine, and comparable with that observed in patients prescribed SSRIs.

**Trial Registration:** ClinicalTrials.gov: NCT05932407

## Abstract 2

This real‐world, retrospective cohort study showed that the incidence of intracranial hemorrhage was low in Japanese patients with depression prescribed vortioxetine, and comparable with that observed in patients prescribed selective serotonin reuptake inhibitors (SSRIs). Nearly half of the reported intracranial hemorrhages occurred within 60 days of vortioxetine or SSRI treatment initiation.

## Graphical abstract

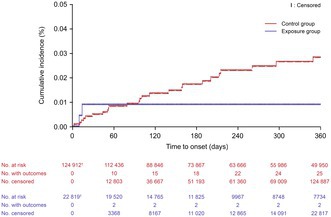

## 1 Introduction

Major depressive disorder (MDD) is a debilitating disease that significantly impairs individuals' lives through its adverse mood, physical symptoms, and cognitive symptoms [1]. In Japan, MDD is considered a common disorder, with lifetime and 12‐month prevalence estimates of 5.7% and 2.7%, respectively [2].

Treatment guidelines recommend the use of selective serotonin reuptake inhibitors (SSRIs) or serotonin and norepinephrine reuptake inhibitors (SNRIs), as well as other antidepressants, for initial treatment of MDD [3, 4]. Antidepressants with several mechanisms of action (e.g., SSRIs, SNRIs, and nonadrenergic and specific serotonergic antidepressants [NaSSAs]), are approved for use in Japan [5]. Vortioxetine is an antidepressant that both selectively inhibits serotonin reuptake and modulates the release of serotinin (via its antagonism of serotonin [5‐hydroxytryptamine; 5‐HT]_3_, 5‐HT_7_, and 5‐HT_1D_ receptors, partial agonism of the 5‐HT_1B_ receptor and agonism of the 5‐HT_1A_ receptor), thus leading to the release of neurotransmitters such as glutamate, dopamine, norepinephrine, acetylcholine, and histamine [6, 7, 8, 9]. In a phase 3 study in Japan, vortioxetine improved Montgomery–Åsberg Depression Rating Scale scores compared with placebo in patients with MDD and was well tolerated [10]. Based on these results and those from several other pivotal studies [10, 11, 12, 13], vortioxetine was approved for use in Japan as an antidepressant in 2019 [14].

Treatment with SSRIs may increase the risk of upper gastrointestinal tract bleeding and is associated with an increased risk of intracranial hemorrhage [15, 16, 17]. This abnormal bleeding risk may be due to the role serotinin plays in platelet aggregation, with SSRIs inhibiting serotinin reuptake by platelets through inhibition of the serotonin transporter that is expressed on these cell fragments [18]. Given that vortioxetine also inhibits serotonin reuptake (by modulating multiple serotonin receptors and blocking the serotonin transporter), and thus presumably inhibits platelet aggregation, its use may also increase bleeding risk.

A recent pharmacovigilance analysis identified a higher proportion of positive signals for hemorrhage‐related adverse events for SSRIs compared with SNRIs such as duloxetine and venlafaxine [19]. Furthermore, a large cohort study in patients with atrial fibrillation using anticoagulants reported that SSRIs were associated with a higher risk of bleeding than SNRIs and tricyclic antidepressants [20]. The inhibition of platelet serotonin uptake by SSRIs may explain the elevated bleeding risk associated with this drug class [21]. Together, these findings support the validity of using SSRIs as a reference drug class for bleeding risk assessments.

Data on the hemorrhagic risk of vortioxetine remain limited. Case reports have described bleeding events such as hemoptysis and uterine bleeding following vortioxetine use [22, 23]. In addition, a pharmacovigilance study assessed bleeding signals associated with vortioxetine [24]. However, the number of published reports and documented cases is small, and no large‐scale studies have confirmed a significant bleeding risk with vortioxetine treatment. Therefore, further investigation is required to clarify whether vortioxetine has unique hemorrhage risks compared with SSRIs.

In a pooled analysis that included two phase 3 studies (NCT01355081 and NCT02389816) conducted in Japanese participants with MDD, and a multinational phase 2/3 study (NCT01255787) that included Japanese participants with MDD, hemorrhage‐related adverse events (AEs) occurred in 0.9% (4/435) of those who received vortioxetine 10 mg once daily, 1.0% (3/313) of those who received vortioxetine 20 mg once daily, and 1.1% (5/436) of those who received placebo [10, 11, 12, 13]. The mean duration of treatment was 53.2 days for vortioxetine 10 mg, 52.7 days for vortioxetine 20 mg, and 52.6 days for placebo [11]. In a long‐term, phase 3 extension study (NCT01395147; extension of NCT01355081), hemorrhage‐related AEs occurred in 3.6% (10/280) of patients (mean treatment duration: 315 days) [11].

In one phase 3 study in Japan (NCT02389816), death was reported in one patient with cerebral hemorrhage that was deemed by the study investigators as likely to be related to vortioxetine treatment, and in one patient with subarachnoid hemorrhage that was considered unrelated to vortioxetine treatment [10]. However, evidence of a causal relationship between vortioxetine treatment and serious intracranial (e.g., cerebral and subarachnoid) hemorrhages remains limited [25].

Hemorrhage was identified by Takeda Pharmaceutical Company Ltd. and the Japanese Pharmaceuticals and Medical Devices Agency as an important potential risk factor for inclusion in the Risk Management Plan for vortioxetine [26]. A post‐marketing commitment was thus made to determine whether vortioxetine has unique, serious hemorrhagic (including cerebral hemorrhage) AE risks compared with conventional SSRIs. As part of this commitment, a post‐marketing study was conducted to assess the incidence of intracranial hemorrhage and serious (intracranial or gastrointestinal) hemorrhage requiring hospitalization in patients with depression who had been prescribed vortioxetine or SSRIs.

## 2 Methods

### 2.1 Study Design

This real‐world, retrospective cohort study in patients with depression utilized the JMDC claims database (JMDC Inc., Tokyo, Japan). Dating from 2005, the JMDC claims database contains health insurance claims data, medical examinations data, and ledger information provided by Japanese health insurance associations. As of November 2023, the JMDC claims database includes data for approximately 17 million people [27]. This study obtained JMDC claims data collected between November 2018 and November 2023.

This study was conducted in compliance with the Japanese Ministerial Ordinance on Good Post‐Marketing Study Practice (GPSP). JMDC claims data are anonymized and fully deidentified; therefore, participant informed consent was not required. Institutional Review Board and Ethics Committee approvals were not required according to the GPSP. The study was registered (ClinicalTrials.gov ID: NCT05932407).

### 2.2 Study Population

This analysis included new users of vortioxetine or SSRIs who were registered in the JMDC claims database. Key inclusion criteria were a diagnosis of depression and a first prescription of vortioxetine or SSRIs (index date) that occurred between November 2019 and November 2022 (see Table S1 for International Classification of Diseases [ICD]‐10 codes and Anatomical Therapeutic Chemical [ATC] drug codes). Patients also had to have an observation time of 6 months before the index date (look‐back period) and no prescription of vortioxetine or SSRIs before the index date. Patients were excluded if during the look‐back period they had a diagnosis of intracranial hemorrhage (see Outcomes and Definitions) or they used vortioxetine and SSRIs concomitantly at the index date.

The prescription period for vortioxetine or SSRIs was defined as the day after the index date to 30 days after the final prescription end date. Vortioxetine or SSRI prescriptions were considered continuous if the interval between prescription periods (gap period) was 30 days or less; prescriptions were considered discontinued for intervals between prescription periods of 31 days or longer. The observation period started 6 months before the index date (look‐back period). The end date of the observation period was defined as the earliest date when one of the following events occurred: intracranial hemorrhage; end of the vortioxetine or SSRI prescription period; switching from vortioxetine to SSRIs, or from SSRIs to vortioxetine; concomitant use of vortioxetine and SSRIs; death; withdrawal from the health insurance association; or end of contract between the health insurance association and JMDC. In the absence of any of these events, the end date of the observation period was 360 days after the index date (completion of the follow‐up period).

### 2.3 Outcomes and Definitions

The primary outcome was the incidence of intracranial hemorrhage in patients treated with vortioxetine (exposure) or SSRIs (control). Secondary outcomes included the time to onset of intracranial hemorrhage and the incidence of serious hemorrhage requiring hospitalization in patients treated with vortioxetine or SSRIs.

The diagnosis of intracranial hemorrhage and serious hemorrhage requiring hospitalization was based on ICD‐10 codes, hospitalization records, and ATC drug codes. Patients who had intracranial hemorrhage (such as a cerebral or subarachnoid hemorrhage requiring hospitalization) were defined as those who required inpatient treatment and met Criteria set 1 and Criteria set 2 (Methods S1). Patients who had serious hemorrhage (intracranial or gastrointestinal) requiring hospitalization were defined as those who met Criteria set 3 and Criteria set 4 (Methods S1).

### 2.4 Statistical Analyses

A preliminary feasibility assessment using the JMDC claims database was conducted before the main study (Results S1). Sample size calculations were based on the formula $n_{T}=\frac{{\left[z_{1-\alpha }\sqrt{\left(k+1\right)\bar{\lambda }\left(1-\bar{\lambda }\right)}+z_{1-\beta }\sqrt{\lambda_{c}\left(1-\lambda_{c}\right)+k\left(\lambda_{c}+\varepsilon \right)\left(1-\lambda_{c}-\varepsilon_{\text{Plan}}\right)}\right]}^{2}}{k\varepsilon^{2}}$ which has been proposed for comparative research in post‐marketing surveillance [28]. The sample sizes required to detect a two‐fold increase in the incidence of intracranial hemorrhage per year assumed an allocation ratio of 7:1 to the control group or exposure group, a two‐sided significance level (α) of 0.05% and 80% power (1 − β = 0.8) to detect a real difference (one‐sided significance level of 0.05). Patient characteristics were summarized using descriptive statistics including frequency (percentage), mean (standard deviation [SD]), and median (interquartile range [IQR]). Standardized mean differences (SMDs) were calculated to compare baseline characteristics between the exposure and control groups; patient characteristics were considered well balanced if the SMD was less than 0.10 [29].

The incidences of intracranial hemorrhage and serious hemorrhage requiring hospitalization were estimated per 10 000 person‐years for exposure and control groups as well as for each SSRI. Crude hazard ratios (HRs) and adjusted hazard ratios (aHRs) with 95% confidence intervals (CIs) were calculated to compare the incidences of intracranial hemorrhage and serious hemorrhage requiring hospitalization between exposure and control groups. A multivariate analysis was used to compute HRs adjusted for age and sex (covariate set [COV] 1), and for antithrombotic drug prescription, nonsteroidal anti‐inflammatory drug (NSAID) prescription, and hypertension (COV2) (Methods S1), which are considered risk factors for intracranial hemorrhage [17, 30, 31, 32, 33]. Time to onset of intracranial hemorrhage was estimated using the Kaplan–Meier method.

A sensitivity analysis was performed to assess the robustness of the primary analysis. The following parameters were altered to examine their effect on the primary analysis: the look‐back period was changed from 6 months to 1 year before the index date to increase the stringency of this criterion with regard to excluding any patients with recurrent intracranial hemorrhage; the gap period and grace period (interval after the final prescription) were changed from 30 days to 90 days to capture drug‐related outcomes potentially occurring over a broader time frame; and to account for potential differences in positive predictability, specificity and sensitivity, the outcome definition for intracranial hemorrhage (Methods S1) was changed from being required to meet Criteria set 1 and Criteria set 2 to only having to meet Criteria set 1, with Criteria set 2 also altered so that ‘within the same hospitalization’ was changed to ‘the same claim ID or the hospitalization claim ID in the following month after the start of hospitalization’, the drug was changed from any route of administration to injection only, and the suspect flag was changed from ‘excluding cases with suspect flags’ to ‘not considered’. In addition to adjusting for COV1 and COV2, a further 21 covariates (see COV3 in Methods S1) were combined to generate one propensity score that was added to the models to compute HRs in the sensitivity analysis.

## 3 Results

### 3.1 Patient Disposition

**FIGURE 1:**
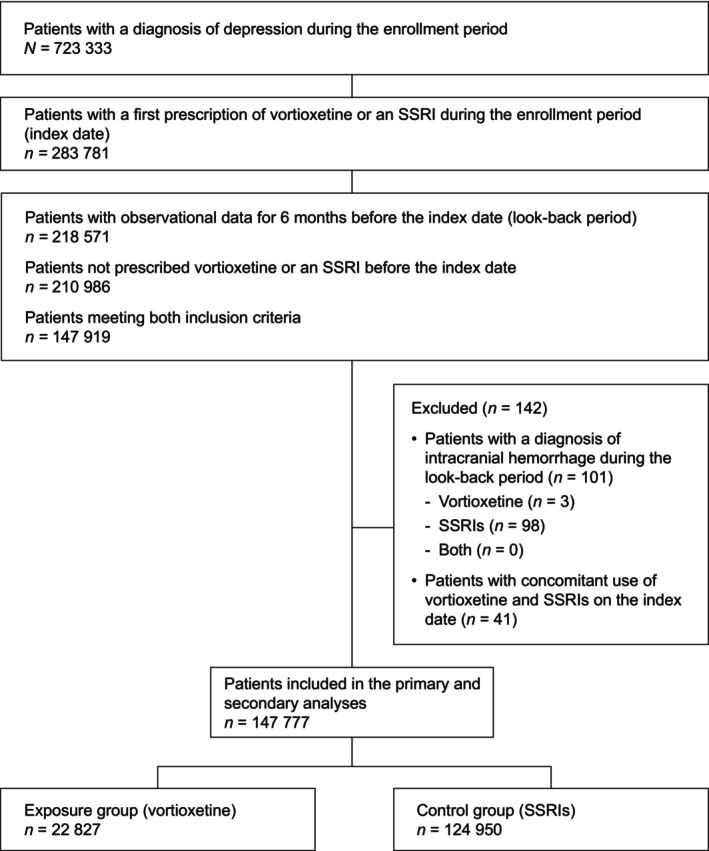
Patient disposition. SSRI, selective serotonin reuptake inhibitor.

Between November 2019 and November 2022, 723 333 patients were diagnosed with depression in Japan, of whom 283 781 were prescribed vortioxetine or SSRIs (Figure 1). Of these patients, 218 571 had observational data for 6 months (look‐back period) before the index date (prescription of vortioxetine or SSRIs) and 210 986 were not prescribed vortioxetine or SSRIs before the index date; 147 919 patients met both criteria. After excluding 142 individuals (101 who were previously diagnosed with intracranial hemorrhage and 41 who received concomitant vortioxetine and SSRIs at the index date), 147 777 were included in the primary and secondary analyses (22 827 prescribed vortioxetine [exposure] and 124 950 prescribed SSRIs [control]) and were followed for up to 360 days.

### 3.2 Patient Characteristics

Patient characteristics were generally well balanced between the exposure and control groups (SMD < 0.10 for most covariates) (Table 1 and Table S2). At the time of the index date, the mean (SD) age was 37.7 (12.5) years in the exposure group and 36.4 (13.3) years in the control group (SMD = 0.10). The proportion of male patients was higher in the exposure group (57.4%) than in the control group (46.5%; SMD = 0.22). In addition, the proportion of patients with concomitant use of atypical antipsychotics was higher in the exposure group (12.0%) than in the control group (7.8%; SMD = 0.14). The SMD between the exposure group and control group was less than 0.10 for all comorbidities at the index date, history of drinking and smoking, and prescription of antithrombotic drugs, NSAIDs, phenothiazine antipsychotics, and tricyclic antidepressants prescribed in the 30 days before the index date.

**TABLE 1:** Patient characteristics.

Characteristic	Control group (*N* = 124 950)	Exposure group (*N* = 22 827)	SMD
Sex, *n* (%)			0.22
Male	58 064 (46.5)	13 113 (57.4)	
Female	66 886 (53.5)	9714 (42.6)	
Age, years^a^			
Mean (SD)	36.4 (13.3)	37.7 (12.5)	0.10
Median (IQR)	36.0 (25.0–47.0)	37.0 (27.0–48.0)	
Age category, *n* (%) years^a^			
0–14	3109 (2.5)	119 (0.5)	−0.16
15–24	25 488 (20.4)	3852 (16.9)	−0.09
25–34	30 823 (24.7)	6038 (26.5)	0.04
35–44	27 532 (22.0)	5353 (23.5)	0.03
45–54	25 815 (20.7)	5139 (22.5)	0.05
55–64	10 517 (8.4)	2083 (9.1)	0.03
65–74	1663 (1.3)	240 (1.1)	−0.03
75+	3 (0.0)	3 (0.0)	0.01
Hypertension, *n* (%)	12 711 (10.2)	2554 (11.2)	0.03
Prescription of antithrombotic drugs, *n* (%)^b^	1814 (1.5)	347 (1.5)	0.01
Prescription of NSAIDs, *n* (%)^b^	10 031 (8.0)	1795 (7.9)	−0.01

*Note:* There were no missing data for covariates other than drinking and smoking history (Table S2). Abbreviations: IQR, interquartile range; NSAID, nonsteroidal anti‐inflammatory drug; SD, standard deviation; SMD, standardized mean difference.

^a^ Age at the index date (day 0).

^b^ Drug prescription from days −30 to −1.

### 3.3 Incidence of Intracranial Hemorrhage

**FIGURE 2:**
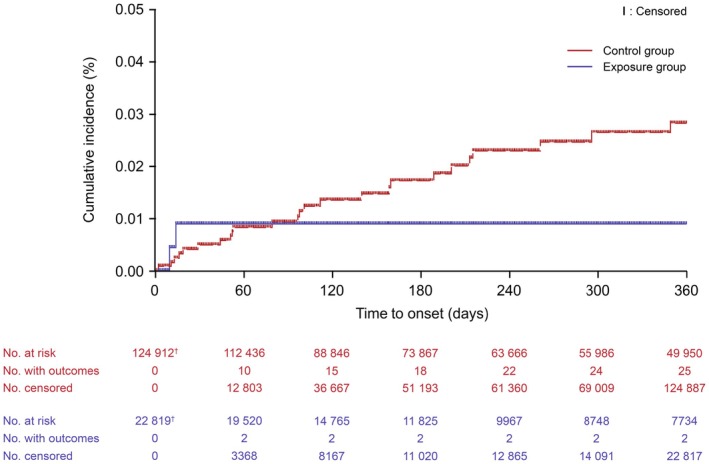
Incidence of intracranial hemorrhage in the exposure and control groups. ^†^Patients whose index date is the same as the end date of the observation period (i.e., patients whose follow‐up period is 0 days) are not included.

Intracranial hemorrhage occurred in two of 22 827 patients prescribed vortioxetine (exposure group) and 25 of 124 950 patients prescribed SSRIs (control group) (Table 2). The incidence of intracranial hemorrhage was 1.5 per 10 000 person‐years in the exposure group after a total follow‐up period of 13 148.4 person‐years, and 3.2 per 10 000 person‐years in the control group after a total follow‐up period of 79 184.9 person‐years (crude HR: 0.5, 95% CI: 0.1–2.0; aHR: 0.5, 95% CI: 0.1–1.9). From the start of the follow‐up period, intracranial hemorrhage occurred within 60 days in two patients in the exposure group and 10 patients in the control group, and within 120 days in 15 patients in the control group (Figure 2).

**TABLE 2:** Incidence of intracranial hemorrhage in the exposure and control groups.

Group	Number of patients	Number of outcomes	Total follow‐up period (person‐years)	Incidence (per 10 000 person‐years)	Crude HR (95% CI)	Adjusted HR^a^ (95% CI)
Control group	124 950	25	79 184.9	3.2	Reference	Reference
Exposure group	22 827	2	13 148.4	1.5	0.5 (0.1–2.0)	0.5 (0.1–1.9)

Abbreviations: CI, confidence interval; COV, covariate set; HR, hazard ratio; NSAID, nonsteroidal anti‐inflammatory drug.

^a^ Adjusted for COV1 (age and sex) and COV2 (antithrombotic drug prescription, NSAID prescription, and hypertension) in a multivariable analysis.

### 3.4 Incidence of Serious Hemorrhage Requiring Hospitalization

Serious hemorrhage (intracranial or gastrointestinal) requiring hospitalization occurred in 36 of 22 827 patients in the exposure group and 248 of 124 950 patients in the control group (Table 3). The incidence of serious hemorrhage requiring hospitalization was 27.4 per 10 000 person‐years in the exposure group after a total follow‐up of 13 134.8 person‐years, and 31.4 per 10 000 person‐years in the control group after a total follow‐up of 79 097.5 person‐years (crude HR: 0.9, 95% CI: 0.6–1.2; aHR: 0.8, 95% CI: 0.6–1.2).

**TABLE 3:** Incidence of serious hemorrhage (intracranial or gastrointestinal) requiring hospitalization in the exposure and control groups.

Group	Number of patients	Number of outcomes	Total follow‐up period (person‐years)	Incidence (per 10 000 person‐years)	Crude HR (95% CI)	Adjusted HR^a^ (95% CI)
Control group	124 950	248	79 097.5	31.4	Reference	Reference
Exposure group	22 827	36	13 134.8	27.4	0.9 (0.6–1.2)	0.8 (0.6–1.2)

^a^ Adjusted for COV1 (age and sex) and COV2 (antithrombotic drug prescription, NSAID prescription, and hypertension) in a multivariable analysis.

### 3.5 Sensitivity Analysis

The robustness of the primary analysis was assessed in the sensitivity analysis; all results were similar to those of the primary analysis. After adjusting for age, sex, antithrombotic drug prescription, NSAID prescription, hypertension, and an additional 21 covariates, the incidence of intracranial hemorrhage in the exposure versus control group was similar to that in the primary analysis (aHR: 0.4, 95% CI: 0.1–1.9) (Table S3).

## 4 Discussion

In this retrospective analysis of patients with depression in the JMDC claims database, the incidence of intracranial hemorrhage was low in patients prescribed vortioxetine and comparable with that observed in patients prescribed SSRIs. Specifically, the incidence of intracranial hemorrhage was 1.5 per 10 000 person‐years for vortioxetine and 3.2 per 10 000 person‐years for SSRIs (aHR: 0.5, 95% CI: 0.1–1.9). In the current study, nearly half (44.4% [12/27]) of the reported intracranial hemorrhages occurred within 60 days of vortioxetine or SSRI treatment initiation.

Although most patient characteristics were well balanced between the exposure and control groups, imbalances were observed for sex, age, and the prescription of atypical antipsychotics. The higher proportion of males in the vortioxetine group compared with the SSRI group follows a similar trend reported in a population‐based study conducted in Italy [34]. Other studies have reported mixed findings when comparing ages between patients receiving vortioxetine and patients receiving SSRI prescriptions [34, 35].

In the current study, both the crude and adjusted HRs for the risk of intracranial hemorrhage in patients treated with vortioxetine versus SSRIs were below 1 and within their respective 95% CIs. These data suggest that the risk of intracranial hemorrhage in patients prescribed vortioxetine does not differ from that in patients prescribed SSRIs. In a UK population‐based cohort study of new users of antidepressants, published in 2017 by Renoux et al., the risk of intracranial hemorrhage with SSRI use was the highest in the first 30 days after the start of treatment, with few events observed thereafter [17]. Similarly, in our study, nearly half of the reported intracranial hemorrhages occurred within 60 days of receiving vortioxetine and SSRI prescriptions. Our study also suggests that the time to onset of intracranial hemorrhage does not differ between patients prescribed vortioxetine and those prescribed SSRIs. Overall, the findings of the current study indicate that the incidence of intracranial hemorrhage is low in patients prescribed vortioxetine and comparable with that observed in patients prescribed SSRIs, although it should be noted that the overall number of events was small.

Our study is the first to evaluate the incidence of intracranial hemorrhage in Japanese patients with depression prescribed SSRIs using the JMDC claims database. A key strength of the large (approximately 17 million people) JMDC claims database is that it is useful for investigating real‐world incidences of rare AEs such as intracranial hemorrhage.

The incidence of intracranial hemorrhage was low in our study despite the use of the large JMDC claims database. In addition, by examining cases of intracranial hemorrhage together with gastrointestinal hemorrhage, which had a higher incidence overall than intracranial hemorrhage alone, our analysis evaluated bleeding risk from multiple angles and was thus highly sensitive and robust.

No major differences were detected between the primary and sensitivity analyses. After adjusting for age, sex, antithrombotic drug prescription, NSAID prescription, hypertension, and an additional 21 covariates, the incidence of intracranial hemorrhage in the exposure versus control group in the sensitivity analysis was similar to the primary analysis. Thus, the results of the primary analysis were confirmed to be robust.

This real‐world study using the JMDC claims database has several limitations that should be considered when interpreting the results. First, patients in the database are employed individuals (and their dependents) living in Japan and are thus not nationally representative [27]. Reflecting this, the proportion of patients aged 65–74 years was only 1.1% in the exposure group and 1.3% in the control group. Therefore, using the JMDC claims database limits the generalizability of our findings to the elderly population, who have an increased risk of intracranial hemorrhage [33]. In addition, the age distribution of patients with depression in the JMDC claims database may differ from the overall Japanese population with depression. Given the underrepresentation of older adults in this study, additional investigation in the elderly population may be required. As the JMDC claims database is based on health insurance claims data, the proportion of employed individuals including those working for relatively large companies is high. Therefore, self‐employed individuals and those working in agricultural and fishing industries are not captured in the database. However, while the proportion of individuals aged more than 65 years is low in the JMDC claims database, the age structure up to 65 years is relatively consistent with Japan's overall population. Second, although patients are highly traceable within the JMDC claims database, they are sometimes lost to follow‐up as a result of withdrawing from a health insurance society, or if JMDC terminates a contract with their health insurance society. Third, the JMDC database uses health insurance claims data as its source. As such, the name of the injury or disease that is entered into insurance claims may differ from the actual diagnosis. Similarly, prescription information does not necessarily reflect whether or not a patient is actually taking their prescribed medication. In addition, claims data lack clinical details (e.g., bleeding tendency, platelet counts and prescriber rationale). Therefore, residual confounding, particularly confounding by indication, remains an important limitation of our study. Finally, data on alcohol consumption and smoking, which are risk factors for intracranial hemorrhage [31, 32], were missing for about 75% of patients in the current study. In the primary and secondary analyses, drinking and smoking history had lower priorities as covariates compared with the use of age, sex, antithrombotic drug prescription, NSAID prescription, and hypertension, and were thus not used in the multivariate analysis. However, drinking and smoking history were used as covariates in the propensity score, and the missing data for these variables are therefore a potential limitation of the sensitivity analysis that assessed the robustness of the primary analysis.

## 5 Conclusion

The incidence of intracranial hemorrhage and serious hemorrhage requiring hospitalization was low in Japanese patients with depression prescribed vortioxetine, and comparable with that observed in patients prescribed SSRIs.

## Author Contributions

**Takeshi Inoue:** data interpretation. **Rie Otake:** conceptualization and design, data acquisition, formal analysis, data interpretation. **Tatsuya Hoshino:** data interpretation. All authors reviewed the manuscript for important intellectual content and approved the final manuscript.

## Funding

This work was supported by Takeda Pharmaceutical Company Ltd, and Lundbeck Japan K.K.

## Ethics Statement

This study was conducted in compliance with the Japanese Ministerial Ordinance on Good Post‐Marketing Study Practice (GPSP). Institutional Review Board and Ethics Committee approvals were not required according to the GPSP Ordinance.

## Consent

No direct patient contact or primary collection of individual human data occurred in this study. JMDC claims data are anonymized and fully deidentified; therefore, participant informed consent was not required.

## Conflicts of Interest

Takeshi Inoue has received personal compensation from Eli Lilly, Janssen Pharmaceutical, Lundbeck, Meiji Seika Pharma, Mitsubishi Tanabe Pharma, Mochida Pharmaceutical, MSD, Nippon Boehringer Ingelheim Co., Takeda Pharmaceutical Company, Viatris Pharmaceuticals Japan Inc., and Yoshitomiyakuhin, has received grants and personal compensation from Eisai, Daiichi Sankyo, Kyowa Pharmaceutical Industry, Otsuka Pharmaceutical, Shionogi, and Sumitomo Pharma, and is a member of the advisory boards for Nippon Boehringer Ingelheim Co., Otsuka Pharmaceutical, Takeda Pharmaceutical Company, and Viatris Pharmaceuticals Japan Inc. Rie Otake and Tatsuya Hoshino are employees of Takeda Pharmaceutical Company Ltd.

## Supporting information


**Table S1:** ICD‐10 codes and ATC drug codes.
**Table S2:** Additional patient characteristics.
**Table S3:** Incidence of intracranial hemorrhage in the propensity score analysis.

## Data Availability

The health insurance data used in this study are available from JMDC Inc. Access to these data is limited and not publicly available as they were used under a license for the current study.

## References

[1] C. Otte , S. M. Gold , B. W. Penninx , et al., “Major Depressive Disorder,” Nature Reviews. Disease Primers 2 (2016): 16065. 10.1038/nrdp.2016.65 27629598

[2] H. Ishikawa , H. Tachimori , T. Takeshima , et al., “Prevalence, Treatment, and the Correlates of Common Mental Disorders in the Mid 2010's in Japan: The Results of the World Mental Health Japan 2nd Survey,” Journal of Affective Disorders 241 (2018): 554–562. 30153639 10.1016/j.jad.2018.08.050

[3] A Committee for Treatment Guidelines of Mood Disorders of the Japanese Society of Mood Disorders , “Guidelines for Treatment of Depression: Depression/Major Depressive Disorder,” (2016) accessed 22 February 2024, https://www.secretariat.ne.jp/jsmd/iinkai/katsudou/data/20190724‐02.pdf. (In Japanese).

[4] R. W. Lam , S. H. Kennedy , C. Adams , et al., “Canadian Network for Mood and Anxiety Treatments (CANMAT) 2023 Update on Clinical Guidelines for Management of Major Depressive Disorder in Adults: Réseau canadien pour les traitements de l'humeur et de l'anxiété (CANMAT) 2023: Mise à jour des lignes directrices cliniques pour la prise en charge du trouble dépressif majeur chez les adultes,” Canadian Journal of Psychiatry 69, no. 9 (2024): 641–687. 38711351 10.1177/07067437241245384 PMC11351064

[5] H. Sakurai , H. Uchida , M. Kato , et al., “Pharmacological Management of Depression: Japanese Expert Consensus,” Journal of Affective Disorders 266 (2020): 626–632. 32056937 10.1016/j.jad.2020.01.149

[6] C. Sanchez , K. E. Asin , and F. Artigas , “Vortioxetine, a Novel Antidepressant With Multimodal Activity: Review of Preclinical and Clinical Data,” Pharmacology & Therapeutics 145 (2015): 43–57. 25016186 10.1016/j.pharmthera.2014.07.001

[7] B. Bang‐Andersen , T. Ruhland , M. Jorgensen , et al., “Discovery of 1‐[2‐(2,4‐Dimethylphenylsulfanyl)phenyl]Piperazine (Lu AA21004): A Novel Multimodal Compound for the Treatment of Major Depressive Disorder,” Journal of Medicinal Chemistry 54, no. 9 (2011): 3206–3221. 21486038 10.1021/jm101459g

[8] A. D'Agostino , C. D. English , and J. A. Rey , “Vortioxetine (Brintellix): A New Serotonergic Antidepressant,” Pharmacy and Therapeutics 40, no. 1 (2015): 36–40. 25628505 PMC4296590

[9] E. Dale , M. Grunnet , A. L. Pehrson , et al., “The Multimodal Antidepressant Vortioxetine May Facilitate Pyramidal Cell Firing by Inhibition of 5‐HT3 Receptor Expressing Interneurons: An in Vitro Study in Rat Hippocampus Slices,” Brain Research 1689 (2018): 1–11. 29274875 10.1016/j.brainres.2017.12.025

[10] T. Inoue , K. Sasai , T. Kitagawa , A. Nishimura , and I. Inada , “Randomized, Double‐Blind, Placebo‐Controlled Study to Assess the Efficacy and Safety of Vortioxetine in Japanese Patients With Major Depressive Disorder,” Psychiatry and Clinical Neurosciences 74, no. 2 (2020): 140–148. 31725942 10.1111/pcn.12956 PMC7027855

[11] M. Osawa , T. Nakajima , S. Fujimoto , Y. Ogino , Y. Moriguchi , and T. Inoue , “Pooled Analysis of Safety Information of Clinical Trials of Vortioxetine Including Japanese Patients With Major Depressive Disorder,” Japanese Journal of Clinical Psychopharmacology 24, no. 5 (2021): 527–545.

[12] A. Nishimura , Y. Aritomi , K. Sasai , T. Kitagawa , and A. R. Mahableshwarkar , “Randomized, Double‐Blind, Placebo‐Controlled 8‐Week Trial of the Efficacy, Safety, and Tolerability of 5, 10, and 20 mg/day Vortioxetine in Adults With Major Depressive Disorder,” Psychiatry and Clinical Neurosciences 72, no. 2 (2018): 64–72. 28858412 10.1111/pcn.12565

[13] T. Inoue , A. Nishimura , K. Sasai , and T. Kitagawa , “Randomized, 8‐Week, Double‐Blind, Placebo‐Controlled Trial of Vortioxetine in Japanese Adults With Major Depressive Disorder, Followed by a 52‐Week Open‐Label Extension Trial,” Psychiatry and Clinical Neurosciences 72, no. 2 (2018): 103–115. 29160598 10.1111/pcn.12623

[14] Takeda and Lundbeck announce Ministry of Health, Labour and Welfare (MHLW) approval of Trintellix in Japan (2019) accessed 16 February 2025, https://www.takeda.com/newsroom/newsreleases/2019/takeda‐and‐lundbeck‐announce‐ministry‐of‐health‐labour‐and‐welfare‐mhlw‐approval‐of‐trintellix‐in‐japan/.

[15] S. O. Dalton , C. Johansen , L. Mellemkjaer , B. Norgard , H. T. Sorensen , and J. H. Olsen , “Use of Selective Serotonin Reuptake Inhibitors and Risk of Upper Gastrointestinal Tract Bleeding: A Population‐Based Cohort Study,” Archives of Internal Medicine 163, no. 1 (2003): 59–64. 12523917 10.1001/archinte.163.1.59

[16] F. J. de Abajo , L. A. Rodriguez , and D. Montero , “Association Between Selective Serotonin Reuptake Inhibitors and Upper Gastrointestinal Bleeding: Population Based Case‐Control Study,” BMJ 319, no. 7217 (1999): 1106–1109. 10531103 10.1136/bmj.319.7217.1106 PMC28262

[17] C. Renoux , S. Vahey , S. Dell'Aniello , and J. F. Boivin , “Association of Selective Serotonin Reuptake Inhibitors With the Risk for Spontaneous Intracranial Hemorrhage,” JAMA Neurology 74, no. 2 (2017): 173–180. 27918771 10.1001/jamaneurol.2016.4529

[18] D. Halperin and G. Reber , “Influence of Antidepressants on Hemostasis,” Dialogues in Clinical Neuroscience 9, no. 1 (2007): 47–59. 17506225 10.31887/DCNS.2007.9.1/dhalperin PMC3181838

[19] X. Zhou , S. Xiang , B. Xu , et al., “A Scientific Approach to Hemorrhage Risk Assessment of SSRIs/SNRIs Utilizing the FAERS Database,” Psychiatry Research 346 (2025): 116388. 39923330 10.1016/j.psychres.2025.116388

[20] I. Y. Shao , J. S. Claxton , P. L. Lutsey , L. Y. Chen , R. F. MacLehose , and A. Alonso , “Association of Type of Antidepressant Initiation With Bleeding Risk in Atrial Fibrillation Patients Taking Oral Anticoagulants,” Drugs ‐ Real World Outcomes 8, no. 3 (2021): 383–391. 34014500 10.1007/s40801-021-00258-3 PMC8324721

[21] D. McFarland , D. Merchant , A. Khandai , et al., “Selective Serotonin Reuptake Inhibitor (SSRI) Bleeding Risk: Considerations for the Consult‐Liaison Psychiatrist,” Current Psychiatry Reports 25, no. 3 (2023): 113–124. 36708455 10.1007/s11920-023-01411-1

[22] K.‐H. Chung , “Rapid Onset of Hemoptysis in a Young Man Treated With Vortioxetine,” Psychiatry and Clinical Neurosciences 75, no. 8 (2021): 266–267. 33966317 10.1111/pcn.13224

[23] L. E. Lee and K. H. Chung , “Vortioxetine‐Induced Bleeding Tendency in a Young Woman With Depression: A Case Report,” Psychiatry and Clinical Psychopharmacology 34, no. 4 (2024): 353–355. 39629743 10.5152/pcp.2024.24927 PMC11744379

[24] B. Micallef , J. M. Dogne , J. Sultana , et al., “An Exploratory Study of the Impact of COVID‐19 Vaccine Spontaneous Reporting on Masking Signal Detection in EudraVigilance,” Drug Safety 46, no. 11 (2023): 1089–1103. 37707778 10.1007/s40264-023-01346-9

[25] D. S. Baldwin , L. Chrones , I. Florea , et al., “The Safety and Tolerability of Vortioxetine: Analysis of Data From Randomized Placebo‐Controlled Trials and Open‐Label Extension Studies,” Journal of Psychopharmacology 30, no. 3 (2016): 242–252. 26864543 10.1177/0269881116628440 PMC4794082

[26] Pharmaceuticals and Medical Devices Agency , “Drug Risk Management Plan for Trintellix Tablets 10 mg and Trintellix Tablets 20 mg,” Updated 28 February 2025, accessed 10 January 2025, https://www.pmda.go.jp/RMP/www/400256/88aafa6c‐ab95‐45f1‐baf5‐7538d4fc07d4/400256_1179060F1028_006RMP.pdf.

[27] JMDC Inc ., “JMDC Claims Database,” accessed 24 November 2023, https://www.jmdc.co.jp/en/jmdc‐claims‐database/.

[28] D. Machin , M. J. Campbell , S. B. Tan , and S. H. Tan , Sample Sizes for Clinical, Laboratory and Epidemiology Studies, 4th ed. (Wiley‐Blackwell, 2018).

[29] M. Mamdani , K. Sykora , P. Li , et al., “Reader's Guide to Critical Appraisal of Cohort Studies: 2. Assessing Potential for Confounding,” BMJ 330, no. 7497 (2005): 960–962. 15845982 10.1136/bmj.330.7497.960 PMC556348

[30] M. D. M. Haag , M. J. Bos , A. Hofman , P. J. Koudstaal , M. M. B. Breteler , and B. H. C. Stricker , “Cyclooxygenase Selectivity of Nonsteroidal Anti‐Inflammatory Drugs and Risk of Stroke,” Archives of Internal Medicine 168, no. 11 (2008): 1219–1224. 18541831 10.1001/archinte.168.11.1219

[31] S. Behr , F. Andersohn , and E. Garbe , “Risk of Intracerebral Hemorrhage Associated With Phenprocoumon Exposure: A Nested Case‐Control Study in a Large Population‐Based German Database,” Pharmacoepidemiology and Drug Safety 19, no. 7 (2010): 722–730. 20582908 10.1002/pds.1973

[32] S. Miyamoto , K. Ogasawara , S. Kuroda , et al., “Japan Stroke Society Guideline 2021 for the Treatment of Stroke,” International Journal of Stroke 17, no. 9 (2022): 1039–1049. 35443847 10.1177/17474930221090347 PMC9615334

[33] S. J. An , T. J. Kim , and B. W. Yoon , “Epidemiology, Risk Factors, and Clinical Features of Intracerebral Hemorrhage: An Update,” Journal of Stroke 19, no. 1 (2017): 3–10. 28178408 10.5853/jos.2016.00864 PMC5307940

[34] M. Di Nicola , B. Dell'Osso , I. Peduto , et al., “Adherence to, and Persistence of, Antidepressant Therapy in Patients With Major Depressive Disorder: Results From a Population‐Based Study in Italy,” Current Neuropharmacology 21, no. 3 (2022): 727–739. 10.2174/1570159X20666220411092813 PMC10207922 35410606

[35] M. Zhao , L. Chang , J. Yu , et al., “A Multicenter Retrospective Study of Antidepressant Use in Outpatient Clinics in China Pre‐ and Post‐COVID,” International Journal of Clinical Pharmacy 46, no. 5 (2024): 1215–1224. 39141181 10.1007/s11096-024-01776-0

