# Supplementary material for: Safety of Vortioxetine in Patients With Depression: A Post‐Marketing Surveillance Study of Intracranial Hemorrhage in a Japanese Health Insurance Claims Database
Source: Neuropsychopharmacol Rep. 2025 Dec 25;46(1):e70077. doi: 10.1002/npr2.70077 (PMC12741241; doi:10.1002/npr2.70077)
Supplement: Supplementary file 1 — Table S1: ICD‐10 codes and ATC drug codes. Table S2: Additional patient characteristics. Table S3: Incidence of intracranial hemorrhage in the propensity score analysis. [file NPR2-46-e70077-s001.docx]

**Supplementary Methods**

**Definition of intracranial hemorrhage**

Patients who experienced intracranial hemorrhage, such as cerebral or subarachnoid hemorrhage, were those who required inpatient treatment and met Criteria sets 1 and 2.

Criteria set 1:

a) International Classification of Diseases [ICD]-10 codes: there are records of ICD-10 codes from any of the following: I60 subarachnoid hemorrhage, I61 intracerebral hemorrhage, I62 other nontraumatic intracranial hemorrhage.^1,2^ Excludes cases with suspect flags.

b) There are records of hospitalization (“hospitalization or diagnosis procedure combination [DPC]” in “claim type”) in the same month as the diagnosis of intracranial hemorrhage, such as cerebral hemorrhage and subarachnoid hemorrhage in a) above.

c) There are records of any of the following tests within the same hospitalization (same “claim identification [ID]”) due to intracranial hemorrhage, such as cerebral hemorrhage and subarachnoid hemorrhage in (b) above: computed tomography (CT), magnetic resonance imaging (MRI), or magnetic resonance angiography (MRA) (category code: E200 computed tomography [CT] [per set], E202 magnetic resonance imaging [MRI] [per set], and E203 computed tomography diagnosis).^1^ The first imaging test performed during the same hospitalization was defined as the onset date of the outcome.

Criteria set 2:

In addition to Criteria set 1, any of the following (a) to (f) is recorded within the same hospitalization (same “claim ID”) due to intracranial hemorrhage, such as cerebral hemorrhage and subarachnoid hemorrhage in criterion 1b above.

1. Anti-edema agents: drug code K01F1 (for osmotherapy).^3^
2. Nitrite and nitrate: drug code C01E.^3^
3. Calcium antagonist: drug code C08.^3^
4. Hemostatic drug: name of ingredient (tranexamic acid [B02A1]).^3^
5. Cerebrovascular spasm prophylactic drugs: name of ingredient (ozagrel sodium [B01C9], fasudil hydrochloride hydrate [C04A1], clazosentan sodium [C04A1]).^3^
6. Intracranial hemorrhage treatment: category codes K145 trepanning ventricular drainage, K147 trepanation, K149 decompressive craniectomy, K164 intracranial hematoma evacuation [performed with craniotomy], K176 cerebral aneurysm inflow vessel clipping [performed with craniotomy], and K178 cerebrovascular surgery.^2,3^

**Definition of serious hemorrhage requiring hospitalization**

Patients who experienced serious hemorrhage (intracranial or gastrointestinal) requiring hospitalization were those who met Criteria sets 3 and 4.

Criteria set 3:

There are records of ICD-10 codes from any of the following (excluding cases with suspect flags): intracranial hemorrhage (I60 subarachnoid hemorrhage, I61 intracerebral hemorrhage, I62 other nontraumatic intracranial hemorrhage); or gastrointestinal hemorrhage (I850 esophageal varices with bleeding, K226 gastro-esophageal laceration-hemorrhage syndrome, K250 gastric ulcer/acute with hemorrhage, K251 gastric ulcer/acute with perforation, K252 gastric ulcer/acute with both hemorrhage and perforation, K253 gastric ulcer/acute without hemorrhage or perforation, K254 gastric ulcer/chronic or unspecified with hemorrhage, K255 gastric ulcer/chronic or unspecified with perforation, K256 gastric ulcer/chronic or unspecified with both hemorrhage and perforation, K260 duodenal ulcer/acute with hemorrhage, K261 duodenal ulcer/acute with perforation, K262 duodenal ulcer/acute with both hemorrhage and perforation, K263 duodenal ulcer/acute without hemorrhage or perforation, K264 duodenal ulcer/chronic or unspecified with hemorrhage, K265 duodenal ulcer/chronic or unspecified with perforation, K266 duodenal ulcer/chronic or unspecified with both hemorrhage and perforation, K270 peptic ulcer, site unspecified/acute with hemorrhage, K284 gastrojejunal ulcer/chronic or unspecified with hemorrhage, K285 gastrojejunal ulcer/chronic or unspecified with perforation, K290 acute hemorrhagic gastritis, K920 hematemesis, K921 melena, K922 gastrointestinal hemorrhage, unspecified).^4^

Criteria set 4:

There are records of hospitalization (“hospitalization or DPC” in “claim type”) in the same month as the diagnosis of intracranial hemorrhage, such as cerebral hemorrhage and subarachnoid hemorrhage or gastrointestinal hemorrhage in Criteria set 3 above. Day 15 of the month of hospitalization for intracranial or gastrointestinal hemorrhage was defined as the onset date of the outcome. This definition was used for secondary outcomes only.

**Covariates**

The following covariate sets (COVs) were used to adjust the patient characteristics of the exposure and control groups:

COV1:

- Age (continuous variable)
- Sex (male, female)

COV2:

- Antithrombotic drug prescription (Yes, No)
- Nonsteroidal anti-inflammatory drug (NSAID) prescription (Yes, No)
- Hypertension (Yes, No)

COV3:

- Prescription of atypical antipsychotics (Yes, No)
- Prescription of phenothiazine antipsychotics (Yes, No)
- Prescription of tricyclic antidepressants (Yes, No)
- Diabetes mellitus (Yes, No)
- Ischemic heart disease (Yes, No)
- Ischemic cerebral infarction (Yes, No)
- Cerebral amyloid angiopathy (Yes, No)
- Cerebral aneurysm (Yes, No)
- Brain tumor (Yes, No)
- Epilepsy (Yes, No)
- Liver disease (Yes, No)
- Renal failure (Yes, No)
- Alcohol dependence (Yes, No)
- Epistaxis (Yes, No)
- Dyslipidemia (Yes, No)
- Obesity (Yes, No)
- Metabolic syndrome (Yes, No)
- Sleep apnea syndrome (Yes, No)
- Peripheral arterial disease (Yes, No)
- Drinking history (Yes, No)
- Smoking history (Yes, No)

All drug prescriptions were from day −30 to day −1 before the index date (day 0) for COV2 and COV3. A category of ‘unknown’ was created for patients missing values for drinking and smoking history for COV3.

In addition, COV1 and COV2 were used for multivariate analysis; COV1, COV2, and COV3 were used for propensity score analysis.

The covariates selected for the current study (age and sex, as well as comorbidities and drug prescriptions) are considered risk factors for intracranial hemorrhage.^3,5-8^ Covariate sets COV1 and COV2 used for the multivariate analysis included important confounding factors and assumed approximately 5 to 10 explanatory variables given that outcomes were expected to occur in approximately 50 to 100 patients.^9^ Covariates selected for COV2 included antithrombotic drugs, as a previous study reported that their concomitant use with SSRIs increases the risk of intracranial hemorrhage, and NSAIDs, which are associated with an increased risk of stroke.^6,7^ Hypertension was also selected as a covariate for COV2 as this comorbidity is a modifiable risk factor for cerebral hemorrhage,^3^ and had the highest prevalence in the preliminary feasibility assessment.

**Supplementary Results**

**Preliminary feasibility assessment**

The preliminary feasibility study using the JMDC claims database showed that the number of patients with depression requiring drug therapy between November 2019 (the launch date of vortioxetine in Japan) and January 2022 was approximately 172 000. The number of patients that satisfied the inclusion and exclusion criteria was approximately 11 000 in the exposure group and 76 000 in the control group. Considering that the enrollment period was up to November 2022, it was estimated that the number of patients for inclusion in the current study would be approximately 14 000 in the exposure group and 101 000 in the control group.

In a study published in 2017 by Renoux C. *et al*. that used the UK’s Clinical Practice Research Datalink, more than 1.36 million adults who started using SSRIs, tricyclic antidepressants (TCAs) or other antidepressants for the first time between 1 January 1995 and 30 June 2014 were followed up for a mean period of 5.8 years.^6^ The study reported that 3036 patients were diagnosed with intracranial hemorrhage with an incidence of 3.8 per 10 000 persons per year, and that the risk of intracranial hemorrhage increased by 17% with SSRIs compared with TCAs.^6^ In addition, the incidence of cerebral hemorrhage has been reported to be 2.4 times higher in Asian individuals than non-Asian individuals.^10^ Therefore, the incidence of intracranial hemorrhage per year in this study was estimated to be 0.09% in the control group. Sample sizes of 13 242 patients in the exposure group and 92 694 patients in the control group were required to detect and an additional 0.09% increase in the incidence of intracranial hemorrhage per year in this study. Moreover, after assuming a 7:1 allocation ratio to the exposure group or control group, the two-sided significance level (α) of 0.05 and the power (1-β) of 0.8 (with the one-sided significance level of 0.05), sample sizes of 10 644 and 74 508 patients were calculated for the control and exposure groups, respectively. These findings support the use of the JMDC claims database in this study.

**Supplementary Tables**

**SUPPLEMENTARY TABLE 1** ICD-10 codes and ATC drug codes.

| **Target drugs** | | | | | | |  |  |  |
| --- | --- | --- | --- | --- | --- | --- | --- | --- | --- |
| **1) Exposure group** | | | | | | |  |  |  |
| Defined by the brand name | | | | | | |  |  |  |
| **ATC code** | | **Ingredient name** | | | | |  |  |  |
| N06A9 | | Vortioxetine hydrobromide | | | | |  |  |  |
|  | | | | | | |  |  |  |
| **2) Control group** | | | | | | |  |  |  |
| Defined by ingredient names | | | | | | |  |  |  |
| **ATC code** | | **Ingredient name** | | | | |  |  |  |
| N06A4 | | Escitalopram oxalate | | | | |  |  |  |
| N06A4 | | Sertraline hydrochloride | | | | |  |  |  |
| N06A4 | | Paroxetine hydrochloride | | | | |  |  |  |
| N06A4 | | Fluvoxamine maleate | | | | |  |  |  |
|  | | | | | | |  |  |  |
| **Depression** | | | | | | |  |  |  |
| Defined by ICD-10 block codes | | | | | | |  |  |  |
| **ICD-10 block code** | | **ICD-10 block name** | | | | |  |  |  |
| F30-F39 | | Mood [affective] disorders | | | | |  |  |  |
|  | | | | | | | | | |
| **Intracranial hemorrhage** | | | | | | | | | |
| Defined by ICD-10 subcategory codes | | | | | | | | | |
| **ICD-10 category code** | | **ICD-10 category name** | **ICD-10 subcategory code** | | | **ICD-10 subcategory name** | | | |
| I60 | | Subarachnoid hemorrhage | I600 | | | Subarachnoid hemorrhage from carotid siphon and bifurcation | | | |
| I60 | | Subarachnoid hemorrhage | I601 | | | Subarachnoid hemorrhage from middle cerebral artery | | | |
| I60 | | Subarachnoid hemorrhage | I602 | | | Subarachnoid hemorrhage from anterior communicating artery | | | |
| I60 | | Subarachnoid hemorrhage | I603 | | | Subarachnoid hemorrhage from posterior communicating artery | | | |
| I60 | | Subarachnoid hemorrhage | I604 | | | Subarachnoid hemorrhage from basilar artery | | | |
| I60 | | Subarachnoid hemorrhage | I605 | | | Subarachnoid hemorrhage from vertebral artery | | | |
| I60 | | Subarachnoid hemorrhage | I606 | | | Subarachnoid hemorrhage from other intracranial arteries | | | |
| I60 | | Subarachnoid hemorrhage | I607 | | | Subarachnoid hemorrhage from intracranial artery, unspecified | | | |
| I60 | | Subarachnoid hemorrhage | I608 | | | Other subarachnoid hemorrhage | | | |
| I60 | | Subarachnoid hemorrhage | I609 | | | Subarachnoid hemorrhage, unspecified | | | |
| I61 | | Intracerebral hemorrhage | I610 | | | Intracerebral hemorrhage in hemisphere, subcortical | | | |
| I61 | | Intracerebral hemorrhage | I611 | | | Intracerebral hemorrhage in hemisphere, cortical | | | |
| I61 | | Intracerebral hemorrhage | I613 | | | Intracerebral hemorrhage in brain stem | | | |
| I61 | | Intracerebral hemorrhage | I614 | | | Intracerebral hemorrhage in cerebellum | | | |
| I61 | | Intracerebral hemorrhage | I615 | | | Intracerebral hemorrhage, intraventricular | | | |
| I61 | | Intracerebral hemorrhage | I616 | | | Intracerebral hemorrhage, multiple localized | | | |
| I61 | | Intracerebral hemorrhage | I618 | | | Other intracerebral hemorrhage | | | |
| I61 | | Intracerebral hemorrhage | I619 | | | Intracerebral hemorrhage, unspecified | | | |
| I62 | | Other nontraumatic intracranial hemorrhage | I620 | | | Nontraumatic subdural hemorrhage | | | |
| I62 | | Other nontraumatic intracranial hemorrhage | I621 | | | Nontraumatic extradural hemorrhage | | | |
| I62 | | Other nontraumatic intracranial hemorrhage | I629 | | | Intracranial hemorrhage (nontraumatic), unspecified | | | |
|  | | | | | | | | | |
| **Gastrointestinal hemorrhage** | | | | | | | | | |
| Defined by ICD-10 subcategory codes | | | | | | | | | |
| **ICD-10 subcategory code** | | **ICD-10 subcategory name** | | | | | | | |
| I850 | | Esophageal varices with bleeding | | | | | | | |
| K226 | | Gastro-esophageal laceration-hemorrhage syndrome | | | | | | | |
| K250 | | Gastric ulcer/acute with hemorrhage | | | | | | | |
| K251 | | Gastric ulcer/acute with perforation | | | | | | | |
| K252 | | Gastric ulcer/acute with both hemorrhage and perforation | | | | | | | |
| K253 | | Gastric ulcer/acute without hemorrhage or perforation | | | | | | | |
| K254 | | Gastric ulcer/chronic or unspecified with hemorrhage | | | | | | | |
| K255 | | Gastric ulcer/chronic or unspecified with perforation | | | | | | | |
| K256 | | Gastric ulcer/chronic or unspecified with both hemorrhage and perforation | | | | | | | |
| K260 | | Duodenal ulcer/acute with hemorrhage | | | | | | | |
| K261 | | Duodenal ulcer/acute with perforation | | | | | | | |
| K262 | | Duodenal ulcer/acute with both hemorrhage and perforation | | | | | | | |
| K263 | | Duodenal ulcer/acute without hemorrhage or perforation | | | | | | | |
| K264 | | Duodenal ulcer/chronic or unspecified with hemorrhage | | | | | | | |
| K265 | | Duodenal ulcer/chronic or unspecified with perforation | | | | | | | |
| K266 | | Duodenal ulcer/chronic or unspecified with both hemorrhage and perforation | | | | | | | |
| K270 | | Peptic ulcer, site unspecified/acute with hemorrhage | | | | | | | |
| K284 | | Gastrojejunal ulcer/chronic or unspecified with hemorrhage | | | | | | | |
| K285 | | Gastrojejunal ulcer/chronic or unspecified with perforation | | | | | | | |
| K290 | | Acute hemorrhagic gastritis | | | | | | | |
| K920 | | Hematemesis | | | | | | | |
| K921 | | Melena | | | | | | | |
| K922 | | Gastrointestinal hemorrhage, unspecified | | | | | | | |
|  | | | | | | | | | |
| **Medical practice (diagnosis)** | | | | | | | | | |
| Defined by the standardized clinical practice code | | | | | | | | | |
| **Medical fee point code** | | **Standardized clinical practice code** | | | **Name of standardized clinical department** | | | | |
| E200 | | 170011710 | | | CT scan (for other than a, b, or c) (per set) | | | | |
| E200 | | 170011810 | | | CT scan (for multi-slice devices with 16 or more and less than 64 slices) (per set) | | | | |
| E200 | | 170012070 | | | Addition for use of contrast media (CT scan) | | | | |
| E200 | | 170012110 | | | Cisternal CT scan (including contrast enhancement) (per set) | | | | |
| E202 | | 170015210 | | | Magnetic resonance imaging (MRI scan) (for other than 1 or 2) (per set) | | | | |
| E203 | | 170015410 | | | Computed tomography diagnosis | | | | |
| E203 | | 170019950 | | | Computed tomography diagnosis taken at another medical institution | | | | |
| E202 | | 170020110 | | | Magnetic resonance imaging (MRI scan) (for 1.5 to < 3 tesla devices) (per set) | | | | |
| E202 | | 170020470 | | | Addition for use of contrast media (MRI scan) | | | | |
| E200 | | 170022290 | | | Subtraction for second and subsequent scans (CT, MRI) (per set) | | | | |
| E200 | | 170027770 | | | Addition for coronary CT scan | | | | |
| E202 | | 170027870 | | | Addition for cardiac MRI scan | | | | |
| E200 | | 170028610 | | | CT scan (for multi-slice devices with 4 or more and less than 16 slices) (per set) | | | | |
| E200 | | 170028770 | | | Addition for traumatic whole-body CT | | | | |
| E200 | | 170033410 | | | CT scan (multi-slice devices with 64 or more slices) (for others) (per set) | | | | |
| E202 | | 170033510 | | | Magnetic resonance imaging (MRI scan) (3 or more tesla devices) (for others) (per set) | | | | |
| E200 | | 170033970 | | | Addition for colorectal CT scan (for multi-slice devices with 64 or more slices) (per set) | | | | |
| E200 | | 170034070 | | | Addition for colorectal CT scan (for multi-slice devices with 16 or more and less than 64 slices) (per set) | | | | |
| E200 | | 170034910 | | | CT scan (multi-slice devices with 64 or more slices) (when performed at a shared facility) (per set) | | | | |
| E202 | | 170035010 | | | Magnetic resonance imaging (MRI scan) (3 or more tesla devices) (when performed at a shared facility) (per set) | | | | |
| E202 | | 170035170 | | | Addition for breast MRI scan | | | | |
| E202 | | 170036170 | | | Addition for pediatric sedation MRI scan | | | | |
| E202 | | 170036270 | | | Addition for head MRI scan | | | | |
| E202 | | 170036370 | | | Addition for pediatric sedation MRI scan (3 or more tesla/shared facility) | | | | |
| E202 | | 170036470 | | | Addition for pediatric sedation MRI scan (3 or more tesla/others) | | | | |
| E202 | | 170036570 | | | Addition for pediatric sedation MRI scan (1.5 to < 3 tesla) | | | | |
| E202 | | 170036630 | | | Addition for pediatric sedation MRI scan (3 or more tesla/shared use/second and subsequent scans) | | | | |
| E202 | | 170036730 | | | Addition for pediatric sedation MRI scan (3 or more tesla/others/second and subsequent scans) | | | | |
| E202 | | 170036830 | | | Addition for pediatric sedation MRI scan (1.5 to < 3 tesla/second and subsequent scans) | | | | |
| E200-2 | | 170036950 | | | Fractional flow reserve computed tomography | | | | |
| E200 | | 170037670 | | | Addition for newborn head injury imaging | | | | |
| E200 | | 170037770 | | | Addition for nursing infant head injury imaging | | | | |
| E200 | | 170037870 | | | Addition for infant head injury imaging | | | | |
| E202 | | 170037970 | | | Addition for whole-body MRI scan | | | | |
| E200 | | 170038710 | | | CT scan (multi-slice devices with 64 or more slices) (when performed at a shared facility) (per set) head injury | | | | |
| E200 | | 170038810 | | | CT scan (multi-slice devices with 64 or more slices) (for others) (per set) head injury | | | | |
| E200 | | 170038910 | | | CT scan (for multi-slice devices with 16 or more and less than 64 slices) (per set) head injury | | | | |
| E200 | | 170039010 | | | CT scan (for multi-slice devices with 4 or more and less than 16 slices) (per set) head injury | | | | |
| E200 | | 170039110 | | | CT scan (for other than a, b, or c) (per set) head injury | | | | |
| E200 | | 170039210 | | | Cisternal CT scan (including contrast enhancement) (per set) head injury | | | | |
| E202 | | 170039410 | | | Magnetic resonance imaging (MRI scan) (3 or more tesla devices) (when performed at a shared facility) (per set) head injury | | | | |
| E202 | | 170039510 | | | Magnetic resonance imaging (MRI scan) (3 or more tesla devices) (for others) (per set) head injury | | | | |
| E202 | | 170039610 | | | Magnetic resonance imaging (MRI scan) (for 1.5 to < 3 tesla devices) (per set) head injury | | | | |
| E202 | | 170039710 | | | Magnetic resonance imaging (MRI scan) (for other than 1 or 2) (per set) head injury | | | | |
| E200 | | 170040210 | | | CT scan (64 or more slices) shared facility (shared diagnostic imaging) | | | | |
| E200 | | 170040310 | | | CT scan (64 or more slices) shared facility/head injury (shared diagnostic imaging) | | | | |
| E200 | | 170040410 | | | CT scan (64 or more slices) (others) (shared diagnostic imaging) | | | | |
| E200 | | 170040510 | | | CT scan (64 or more slices) (others) head injury (shared diagnostic imaging) | | | | |
| E200 | | 170040610 | | | CT scan (16 or more and less than 64 slices) (shared diagnostic imaging) | | | | |
| E200 | | 170040710 | | | CT scan (16 or more and less than 64 slices) head injury (shared diagnostic imaging) | | | | |
| E200 | | 170040810 | | | CT scan (4 or more and less than 16 slices) (shared diagnostic imaging) | | | | |
| E200 | | 170040910 | | | CT scan (4 or more and less than 16 slices) head injury (shared diagnostic imaging) | | | | |
| E200 | | 170041010 | | | CT scan (other than a, b, or c) (shared diagnostic imaging) | | | | |
| E200 | | 170041110 | | | CT scan (other than a, b, or c) head injury (shared diagnostic imaging) | | | | |
| E200 | | 170041210 | | | Cisternal CT scan (including contrast enhancement) (shared diagnostic imaging) | | | | |
| E200 | | 170041310 | | | Cisternal CT scan (including contrast enhancement) head injury (shared diagnostic imaging) | | | | |
| E202 | | 170041410 | | | MRI scan (3 or more tesla) shared facility (shared diagnostic imaging) | | | | |
| E202 | | 170041510 | | | MRI scan (3 or more tesla) (others) (shared diagnostic imaging) | | | | |
| E202 | | 170041610 | | | MRI scan (1.5 to < 3 tesla) (shared diagnostic imaging) | | | | |
| E202 | | 170041710 | | | MRI scan (other than 1 or 2) (shared diagnostic imaging) | | | | |
|  | | | | | | | | | |
| **Medical practice (treatment)** | | | | | | | | | |
| Defined by the standardized clinical practice code | | | | | | | | | |
| **Medical fee point code** | | **Standardized clinical practice code** | | | **Name of standardized clinical department** | | | | |
| K145 | | 150066210 | | | Trepanning ventricular drainage | | | | |
| K145-2 | | 150426210 | | | Subcutaneous cerebrospinal fluid reservoir placement | | | | |
| K147 | | 150067110 | | | Trepanation | | | | |
| K147-2 | | 150411910 | | | Intracranial monitoring device insertion | | | | |
| K149 | | 150067410 | | | Decompressive craniectomy (for others) | | | | |
| K149 | | 150335610 | | | Decompressive craniectomy (for Chiari malformation and syringomyelia) | | | | |
| K149-2 | | 150397510 | | | Posterior fossa decompression | | | | |
| K164 | | 150069510 | | | Intracranial hematoma evacuation (performed with craniotomy) (epidural) | | | | |
| K164 | | 150069610 | | | Intracranial hematoma evacuation (performed with craniotomy) (subdural) | | | | |
| K164 | | 150069710 | | | Intracranial hematoma evacuation (performed with craniotomy) (intracerebral) | | | | |
| K164-3 | | 150069850 | | | Cerebrovascular embolectomy | | | | |
| K164-3 | | 150069950 | | | Cerebrovascular thrombectomy | | | | |
| K164-2 | | 150248250 | | | Chronic subdural hematoma irrigation/evacuation (with trepanation) | | | | |
| K164-2 | | 150273410 | | | Perforation and irrigation for chronic subdural hematoma | | | | |
| K164-4 | | 150335710 | | | Stereotactic intracerebral hematoma evacuation | | | | |
| K164-5 | | 150372310 | | | Endoscopic intracerebral hematoma evacuation | | | | |
| K176 | | 150243610 | | | Cerebral aneurysm inflow vessel clipping (performed with craniotomy) (1 site) | | | | |
| K176 | | 150243710 | | | Cerebral aneurysm inflow vessel clipping (performed with craniotomy) (2 or more sites) | | | | |
| K178 | | 150254910 | | | Cerebrovascular surgery (1 site) | | | | |
| K178-2 | | 150273510 | | | Percutaneous cerebral angioplasty | | | | |
| K178-3 | | 150301110 | | | Percutaneous selective cerebral thrombolysis/embolus removal (for intracranial cerebral blood vessels) | | | | |
| K178-3 | | 150301210 | | | Percutaneous selective cerebral thrombolysis/embolus removal (for cervical cerebral vessels [internal carotid artery, vertebral artery]) | | | | |
| K178 | | 150344410 | | | Cerebrovascular surgery (2 or more sites) | | | | |
| K178 | | 150355410 | | | Cerebrovascular surgery (using a cerebrovascular stent) | | | | |
| K178-4 | | 150372510 | | | Percutaneous cerebral thrombectomy | | | | |
| K178-5 | | 150380850 | | | Percutaneous cerebrovascular stent placement | | | | |
|  | | | | | | | | | |
| **Drugs (treatment)** | | | | | | | | | |
| Defined by an ATC second level code “C08,” an ATC third level code “C01E,” and an ATC fourth level code “K01F1” | | | | | | | | | |
| **ATC second level code** | | **ATC second level name** | | | **ATC third level code** | | | **ATC third level name** | **ATC fourth level code** |
| C01 | | Cardiac medications | | | C01E | | | Nitrite and nitrate | C01E- |
| C08 | | Calcium antagonists | | | C08A | | | Plain calcium antagonists | C08A- |
| K01 | | Intravenous fluids (100 mL or more) | | | K01F | | | Osmotherapy fluid | K01F1 |
|  |  | | | | | | | |  |
| Defined by ingredient names | | | | | | | | |  |
| **ATC code** | | **Ingredient name** | | |  | | |  |  |
| B02A1 | | Tranexamic acid | | |  | | |  |  |
| B01C9 | | Ozagrel sodium | | |  | | |  |  |
| C04A1 | | Fasudil hydrochloride hydrate | | |  | | |  |  |
| C04A1 | | Clazosentan sodium | | |  | | |  |  |
|  | | | | | | | | | |
| Defined by a dosage form large category code “50” | | | | | | | | |  |
| **Column name** | | **Code** | | **Category** |  | | | | |
| Dosage form large category name | | 50 | | Injectable drugs |  | | | | |
|  | | | | | | | | | |
| **Covariates** | | | | | | | | | |
| Defined by ICD-10 categories | | | | | | | | | |
| **Complication** | | | | **ICD-10 classification** | | | | | |
| Diabetes mellitus | | | | [E10-E14] Diabetes mellitus | | | | | |
| Hypertension | | | | [I10-I15] Hypertensive diseases | | | | | |
| Ischemic heart disease | | | | [I20-I25] Ischemic heart diseases | | | | | |
| Ischemic cerebral infarction | | | | [I63] Cerebral infarction [I64] Stroke, not specified as hemorrhage or infarction | | | | | |
| Cerebral amyloid angiopathy | | | | [E859] Amyloidosis, unspecified | | | | | |
| Cerebral aneurysm | | | | [I671] Cerebral aneurysm, nonruptured | | | | | |
| Brain tumor | | | | [C71] Malignant neoplasm of brain | | | | | |
| Epilepsy | | | | [G40] Epilepsy | | | | | |
| Liver disease | | | | [K70-K77] Diseases of liver, [B15-B19] Viral hepatitis | | | | | |
| Renal failure | | | | [N17-N19] Renal failure | | | | | |
| Alcohol dependence | | | | [F10] Mental and behavioral disorders owing to use of alcohol | | | | | |
| Epistaxis | | | | [R040] Epistaxis | | | | | |
| Dyslipidemia | | | | [E785] Hyperlipidemia, unspecified | | | | | |
| Obesity | | | | [E66] Obesity | | | | | |
| Metabolic syndrome | | | | [E889] Metabolic disorder, unspecified | | | | | |
| Sleep apnea syndrome | | | | [G473] Sleep apnea | | | | | |
| Peripheral arterial disease | | | | [I739] Peripheral vascular disease, unspecified | | | | | |
|  | | | | | | | | | |
| Defined by ATC classification | | | | | | | | | |
| **Concomitant medication** | | | | **ATC classification** | | | | | |
| Antithrombotic drugs | | | | [B01] Antithrombotic drugs | | | | | |
| NSAID | | | | [M01A] Nonsteroidal anti-inflammatory and antirheumatic drugs | | | | | |
| Atypical antipsychotics | | | | [N05A1] Atypical antipsychotics | | | | | |
| Phenothiazine antipsychotics | | | | [N05A9] Typical antipsychotics | | | | | |
| Tricyclic antidepressants | | | | [N06A9] All other antidepressants  ^†^However, drugs (ingredient names) other than following tricyclic antidepressants are excluded  Tetracyclic antidepressants: setiptiline maleate, mianserin hydrochloride, and maprotiline hydrochloride  Other antidepressants: trazodone hydrochloride and vortioxetine hydrobromide  NaSSA: mirtazapine | | | | | |

^†^As of May 2024.

ATC, Anatomical Therapeutic Chemical; CT, computed tomography; ICD, International Classification of Diseases; MRI, magnetic resonance imaging; NaSSA, noradrenaline and specific serotonergic antidepressant; NSAID, nonsteroidal anti-inflammatory drug.

**SUPPLEMENTARY TABLE 2** Additional patient characteristics.

| **Characteristic, n (%)** | **Control group**  *N* = 124 950 | **Exposure group**  *N* = 22 827 | **SMD** |
| --- | --- | --- | --- |
| **Diabetes mellitus** | 27 927 (22.4) | 5666 (24.8) | 0.06 |
| **Ischemic heart disease** | 6406 (5.1) | 1112 (4.9) | −0.01 |
| **Ischemic cerebral infarction, *n* (%)** | 3056 (2.4) | 551 (2.4) | 0.00 |
| **Cerebral amyloid angiopathy, *n* (%)** | 17 (0.0) | 1 (0.0) | −0.01 |
| **Cerebral aneurysm, *n* (%)** | 544 (0.4) | 92 (0.4) | −0.01 |
| **Brain tumor, *n* (%)** | 48 (0.0) | 10 (0.0) | 0.00 |
| **Epilepsy, *n* (%)** | 4283 (3.4) | 1040 (4.6) | 0.06 |
| **Liver disease, *n* (%)** | 24 294 (19.4) | 4950 (21.7) | 0.06 |
| **Renal failure, *n* (%)** | 1387 (1.1) | 255 (1.1) | 0.00 |
| **Alcohol dependence, *n* (%)** | 696 (0.6) | 163 (0.7) | 0.02 |
| **Epistaxis, *n* (%)** | 389 (0.3) | 55 (0.2) | −0.01 |
| **Dyslipidemia, *n* (%)** | 15 202 (12.2) | 3251 (14.2) | 0.06 |
| **Obesity, *n* (%)** | 719 (0.6) | 170 (0.7) | 0.02 |
| **Metabolic syndrome, *n* (%)** | 37 (0.0) | 5 (0.0) | 0.00 |
| **Sleep apnea syndrome, *n* (%)** | 2217 (1.8) | 571 (2.5) | 0.05 |
| **Peripheral arterial disease, *n* (%)** | 767 (0.6) | 147 (0.6) | 0.00 |
| **Drinking history, *n* (%)**^†^ |  |  |  |
| Yes | 12 141 (9.7) | 2628 (11.5) | 0.06 |
| Unknown | 99 580 (79.7) | 17 541 (76.8) | −0.07 |
| **Smoking history, *n* (%)**^‡^ |  |  |  |
| Yes | 7604 (6.1) | 1702 (7.5) | 0.05 |
| Unknown | 90 720 (72.6) | 15 802 (69.2) | −0.07 |
| **Prescription of atypical antipsychotics, *n* (%)**^§^ | 9749 (7.8) | 2728 (12.0) | 0.14 |
| **Prescription of phenothiazine antipsychotics, *n* (%)**^§^ | 1735 (1.4) | 399 (1.7) | 0.03 |
| **Prescription of tricyclic antidepressants, *n* (%)**^§^ | 2223 (1.8) | 692 (3.0) | 0.08 |

There were no missing data for covariates other than drinking and smoking history.

^†^Patients who consumed alcohol daily or occasionally were categorized as ‘yes’ and those who consumed alcohol rarely were categorized as ‘no’.

^‡^Patients who habitually smoked cigarettes were categorized as ‘yes’ and those who did not were categorized as ‘no’.

^§^Drug prescription from days −30 to −1.

SMD, standardized mean difference.

**SUPPLEMENTARY TABLE 3** Incidence of intracranial hemorrhage in the propensity score analysis.

| **Group** | **Number of patients** | **Number of outcomes** | **Total follow-up period (person-years)** | **Incidence (per 10 000 person-years)** | **Crude HR  (95% CI)** | **Adjusted HR^†^  (95% CI)** |
| --- | --- | --- | --- | --- | --- | --- |
| Control group | 124 950 | 25 | 79 184.9 | 3.2 | Reference | Reference |
| Exposure group | 22 827 | 2 | 13 148.4 | 1.5 | 0.5 (0.1–2.0) | 0.4 (0.1–1.9) |

^†^Adjusted for COV1 (age and sex), COV2 (antithrombotic drug prescription, NSAID prescription, and hypertension) and COV3 (an additional 21 covariates) by propensity score.

CI, confidence interval; COV, covariate set; HR, hazard ratio.

**References**

1. Sacco RL, Kasner SE, Broderick JP, Caplan LR, Connors JJ, Culebras A, et al. An updated definition of stroke for the 21st century: a statement for healthcare professionals from the American Heart Association/American Stroke Association. Stroke. 2013;44(7):2064-2089.

2. Fujihara K, Yamada-Harada M, Matsubayashi Y, Kitazawa M, Yamamoto M, Yaguchi Y, et al. Accuracy of Japanese claims data in identifying diabetes-related complications. Pharmacoepidemiol Drug Saf. 2021;30(5):594-601.

3. Miyamoto S, Ogasawara K, Kuroda S, Itabashi R, Toyoda K, Itoh Y, et al. Japan Stroke Society Guideline 2021 for the Treatment of Stroke. Int J Stroke. 2022;17(9):1039-1049.

4. Iwagami M, Tomlinson LA, Mansfield KE, Douglas IJ, Smeeth L, Nitsch D. Gastrointestinal bleeding risk of selective serotonin reuptake inhibitors by level of kidney function: A population-based cohort study. Br J Clin Pharmacol. 2018;84(9):2142-2151.

5. Behr S, Andersohn F, Garbe E. Risk of intracerebral hemorrhage associated with phenprocoumon exposure: a nested case-control study in a large population-based German database. Pharmacoepidemiol Drug Saf. 2010;19(7):722-730.

6. Renoux C, Vahey S, Dell'Aniello S, Boivin JF. Association of selective serotonin reuptake inhibitors with the risk for spontaneous intracranial hemorrhage. JAMA Neurol. 2017;74(2):173-180.

7. Haag MDM, Bos MJ, Hofman A, Koudstaal PJ, Breteler MMB, Stricker BHC. Cyclooxygenase selectivity of nonsteroidal anti-inflammatory drugs and risk of stroke. Arch Intern Med. 2008;168(11):1219-1224.

8. An SJ, Kim TJ, Yoon BW. Epidemiology, risk factors, and clinical features of intracerebral hemorrhage: an update. J Stroke. 2017;19(1):3-10.

9. Vittinghoff E, McCulloch CE. Relaxing the rule of ten events per variable in logistic and Cox regression. Am J Epidemiol. 2006;165(6):710-718.

10. Hori M. Learn about characteristics of Japanese from large-scale clinical studies. Heart. 2015;47(2):124-129.
